# Combination of cyclophosphamide and cytarabine as induction regimen for newly diagnosed adult acute myeloid leukemia

**DOI:** 10.1002/jha2.76

**Published:** 2020-08-11

**Authors:** Qingguo Liu, Hongye Gao, Junfan Li, Yimin Hu, Lihua Wu, Xin Zhao, Shangzhu Li

**Affiliations:** ^1^ Department of Internal Medicine State Key Laboratory of Experimental Hematology National Clinical Research Center for Blood Diseases Institute of Hematology & Blood Diseases Hospital Chinese Academy of Medical Sciences & Peking Union Medical College Tianjin 300020 China

**Keywords:** acute myeloid leukemia, cyclophosphamide, cytarabine, induction chemotherapy

## Abstract

Optimizing the induction therapy of acute myeloid leukemia (AML) may improve the remission rate and reduce the risk of relapse, thereby extend survival. Cyclophosphamide (CTX) shows benefit in treating relapsed and refractory AML patients, but it has not been reported in first‐line induction regimens. To assess the efficacy and safety of CTX and moderate‐dosage cytarabine (Ara‐C) as induction chemotherapy in newly diagnosed adult AML, 40 patients were enrolled to receive CTX (20 mg/kg/d) for 4 consecutive days and Ara‐C for 3 (1 g/m^2^ q12h, CA4+3) or 5 (1 g/m^2^ qd, CA4+5) days. With one course of induction chemotherapy, the overall response rate and the complete remission rate (CR) was 82.5% (33/40) and 77.5% (31/40), respectively. The expected 5 years overall survival and relapse‐free survival was 64% in patients experienced CR and fulfilled consolidation therapy. The neutrophil and platelet recovery time were 17 (range, 10–20) days and 16.5 (range, 12–30) days in the CA4+3 group, faster than that of 20 (16‐36) days and 20 (14‐36) days in the CA4+5 group (*P* = .006 and *P* = .006). The cyclophosphamide and cytarabine (CA) regimen was generally safe and had reversible adverse effects. The patients who failed to respond to the CA regimen did not benefit from a second course of other traditional induction chemotherapy either. In conclusion, the combined regimen of CTX and Ara‐C represents a promising therapeutic approach to induce the first CR of newly diagnosed adult AML.

## 1 INTRODUCTION

Acute myeloid leukemia (AML), a malignant disorder of hematopoietic stem cells, accounts for approximately 80% of adult acute leukemia [1]. AML outcomes have been improved every decade since 1960 due to advances in supportive care, the use of higher doses of cytosine arabinoside (Ara‐C), and daunorubicin. The achievement of complete remission (CR) during induction is imperative and is considered to be one of the most important factors in improving prognosis [2, 3, 4, 5]. A better initial cytoreduction reduces or delays the risk of subsequent relapse [4, 6]. Regimens producing CR rates higher than 60% showed better overall survival (OS) than those inducing CR rates in < 60% of patients (53 vs 28 wks). Therefore, it is important to achieve CR as soon as possible for a newly diagnosed leukemia patient.

Ara‐C combined with one of anthracyclines has been the standard induction therapy to AML. It is possible to achieve a remission rate up to 80% with one or more courses of induction therapy [7, 8, 9]. Researchers tried to improve patients’ response through dose intensification [10, 11], and incorporation of molecular targeted agents like Fms‐like tyrosine kinase 3 (FLT3) inhibitors [12] or Isocitrate dehydrogenase (IDH) inhibitors [13], but the CR rate of induction course has not been improved significantly [14, 15]. Moreover, anthracycline containing regimens are associated with the risk of long‐term cardiotoxicity [16]. Undoubtedly, finding some other chemotherapy regimens that may improve efficacy as well as lower toxicity will shed light on AML treatment.

Cyclophosphamide (CTX) has been used successfully as salvage regimens for refractory/relapsed AML. The overall response rate (ORR) of adult refractory/relapsed AML is up to 56.7% using medium‐/high‐dose Ara‐C combined with CTX [17]. A salvage MAC protocol (mitoxantrone + Ara‐C + CTX) also achieved ORR of 70% in AML patients with primary induction failure or relapse [18]. However, the efficacy of CTX in the first‐line treatment of AML has not been reported. The protocol containing CTX can be a good alternative if it can achieve a CR rate similar to that of the Daunorubicin (DNR) scheme. Thus, we designed the study to explore the efficacy and safety of CTX combined with Ara‐C for newly diagnosed AML.

## 2 PATIENTS AND METHODS

### 2.1 Patients

Patients that newly diagnosed as de novo AML between January 2014 and April 2019 were enrolled in the study after written informed consent was obtained following the Declaration of Helsinki. The protocol was approved by the Institutional Review Board and the Institutional Ethics Committee of Institute of Hematology & Blood Diseases Hospital, Chinese Academy of Medical Sciences & Peking Union Medical College. The diagnosis and classification of AML were based on the criteria of the 2016 revision of the World Health Organization Classification of Myeloid Neoplasms and Acute Leukemia [19]. Patients were assessed to a risk group according to National Comprehensive Cancer Network (NCCN) classification.

### 2.2 Treatment protocols

CTX was administered at 20 mg/kg/d for 4 consecutive days in both arms. Ara‐C was administered at 1 g/m^2^ every 12 hours for 3 consecutive days in CA4+3 protocol, and 1 g/m^2^ once per day for 5 consecutive days in CA4+5 protocol. The patients were randomly assigned to CA4+3 or CA4+5 protocols at a ratio of 1:1. Patients with intermediate or poor prognosis were recommended to allogeneic Hematopoietic stem cell transplantation (HSCT) (Allo‐HSCT) once achieved first CR. According to NCCN guidelines, patients who refused Allo‐HSCT entered three courses of consolidation chemotherapy including at least two courses of high‐dose Ara‐C (2 g/m^2^ q12h) or standard‐dose Ara‐C combined with an anthracycline. For patients who failed to achieve CR after the initial induction chemotherapy, a standard dosage induction therapy (DA3+7, daunorubicin 45 mg/m^2^, Ara‐C 100 mg/m^2^) was applied.

### 2.3 Efficacy evaluation

Bone marrow was collected between 28 and 35 days from the date of discontinuation of chemotherapy. Bone marrow morphological detection, cytogenetics, and genetic detection were performed to evaluate response. Flow cytometry was performed to evaluate minimal residual disease. Responses were defined as in Ref. [20]: CR as < 5% of bone marrow blasts with normal peripheral blood counts (neutrophils ≥1.0 × 10^9^/L, platelets ≥100 × 10^9^/L); CR with incomplete hematologic recovery (CRi, neutrophils < 1.0 × 10^9^/L, and/or platelets < 100 × 10^9^/L)); partial remission (PR) as bone marrow blasts 5‐25%; No response (NR) as bone marrow blast > 25% and a decrease of pretreatment bone marrow blast percentage by less than 50%. ORR was calculated as the sum of CR, CRi, and PR. Neutrophil recovery was defined as days from the course start of induction therapy to neutrophil recovered to > 0.5 × 10^9^/L. Platelet recovery was defined as days from the course start of chemotherapy to platelets recovered to > 20 × 10^9^/L for twice evaluation without platelet transfusion.

### 2.4 Safety evaluation

Toxicity was assessed using National Cancer Institute Common Terminology Criteria for Adverse Events version 5.0 (https://ctep.cancer.gov/protocolDevelopment/electronic_applications/ctc.htm#ctc_50).

### 2.5 Statistical analysis

SPSS software (SPSS 25.0, Chicago, IL, USA) was used for statistical analysis. Comparisons between categorical variables were performed with the χ2 test or Fisher's exact test. The differences between continuous variables were compared using the *t‐*test or the Mann‐Whitney *U* test. Survival was evaluated by the Kaplan‐Meier method, and the statistical differences were evaluated using the log‐rank test. *P* < .05 was considered to be a statistically significant difference.

## 3 RESULTS

### 3.1 Patient characteristics

**FIGURE 1:**
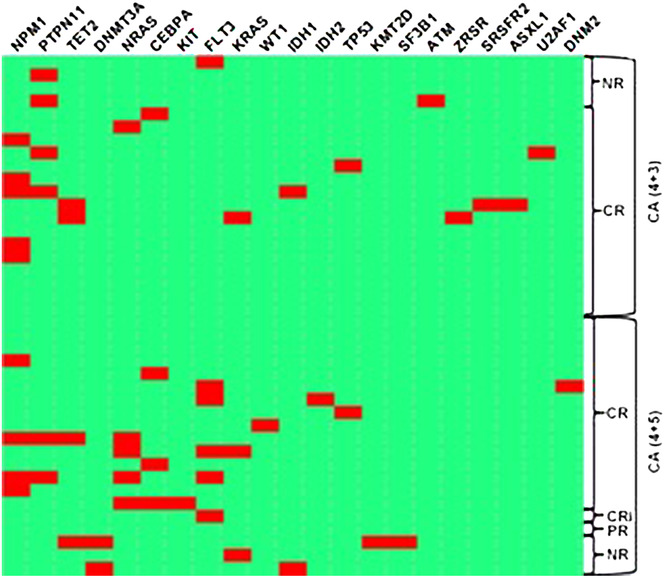
Heatmap of genetic abnormalities. CR: Complete remission; NR: No response; PR: partial remission; CRi: CR with incomplete hematologic recovery

Forty patients at a median age of 44.5 (range 16‐69) years old were enrolled in this study, with 20 patients assigned to the CA4+3 group and 20 assigned to the CA4+5 group. There were 28 male patients (70%) and 12 females. Four patients (10%) carried core‐binding factor alternations. Recurrent genetic abnormalities of *NPM1* and *CEBPA* were found in 13 patients. Seventeen patients were classified as AML not otherwise specified (AML NOS). AML with myelodysplasia‐related changes, *GATA2*, and *MLLT10* abnormalities were detected in other six patients, respectively. There was no significant difference of disease classification in two groups (Table 1). Mutations in AML‐related genes were analyzed. *FLT3*, *ASXL1*, or *TP53* mutations in AML Not otherwise specified (NOS) were detected in three patients of the CA4+3 group, while *FLT3^high^* mutations without *CBFB‐MYH11* or *NPM1* in four patients and TP53 mutations in one patient with AML NOS were identified in the CA4+5 group (Figure 1). According to the NCCN Risk Stratification, 14 patients were assigned to the favorable group, 13 to the intermediate group, and 13 to the adverse group.

**TABLE 1:** Comparison of clinical characteristics between CA4+3 and CA4+5

Characteristics	CA4+3	CA4+5	*P* Value
Age (years)			
Median (range)	50 (24‐69)	38 (16–68)	.261
Gender			.490
Male (%)	13 (65)	15 (75)	
Female (%)	7 (35)	5 (25)	
WBC count (× 10^9^/L)			
Median (range)	12.56 (1.43‐89.96)	9.925 (1–146)	.583
Blast percentage			
Median (range)	55.75 (22. 5–88.5)	60.75 (26.0–90.0)	.529
Infection and/or fever (%)	11 (55)	10 (50)	.752
Classification (%)			
NOS	7 (35)	10 (50)	
*RUNX1‐RUNX1T1*	2 (10)	0 (0)	
*CBFB‐MYH11*	1 (5)	1 (5)	
*GATA2, MECOM (EVI1)*	1 (5)	0 (0)	
*NPM1*	5 (25)	4 (20)	
*CEBPA*	1 (5)	3 (15)	
MLLT10	2 (10)	0 (0)	
Myelodysplasia‐related	1 (5)	2 (10)	
NCCN Risk Stratification (%)			.208
Favorable	9 (45)	5 (25)	
Intermediate	4 (20)	9 (45)	
Adverse	7 (35)	6 (30)	

The bibliography characteristics matched well between the CA4+3 and CA4+5 groups, with comparable age, gender, initial white blood cell (WBC) counts, blast percentage in bone marrow at diagnosis, and NCCN categories.

### 3.2 Response to induction therapy

All 40 patients survived to evaluate the response of induction chemotherapy. The ORR was 82.5% (33/40) after the first course of cyclophosphamide and cytarabine (CA) treatment. Thirty‐one patients achieved CR (77.5%), including 16 patients in the CA4+3 group and 15 patients in the CA4+5 group (80% vs 75%, *P *= .705). One patient in the CA4+5 group achieved CRi, and another patient achieved PR. The four patients who did not respond to the CA4+3 protocol belonged to the adverse risk group. One of three nonresponders in the CA4+5 group was in the adverse risk group. Factors‐affecting CR and ORR were assessed by univariate and multivariate logistic regression. There was no difference of a response rate between the two groups related to gender, age, initial WBC count, classification, cytogenetic, and NCCN risk groups.

Nine patients who did not achieve CR proceeded to a course of traditional induction chemotherapy. Three patients died during the marrow suppression period due to infections or hemorrhage. The remaining six patients still did not achieve CR after the second course and were verified as primary refractory leukemia.

### 3.3 Long‐term outcomes

The last follow‐up was in December 2019, and all the patients were evaluable. By the end of follow‐up, 15 patients were alive and remained CR. The median OS was 16 months (range 1.5‐66 months), with 23.5 months in CA4+3 and 14.5 months in the CA4+5 group, respectively. The expected 5 years survival was 35% within the whole study.

**FIGURE 2:**
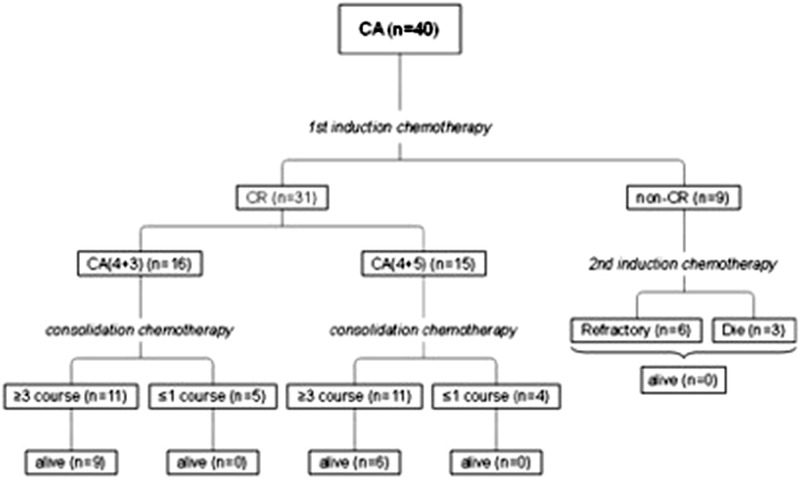
The survival of patients based on consolidation depth

By the end of follow‐up, all the nine patients that did not achieve CR after CA induction died (Figure 2). Among the 31 CR patients, nine patients did not complete the scheduled consolidation regimen due to financial reasons or patients’ decisions, and none was alive by the end of follow‐up.

**FIGURE 3:**
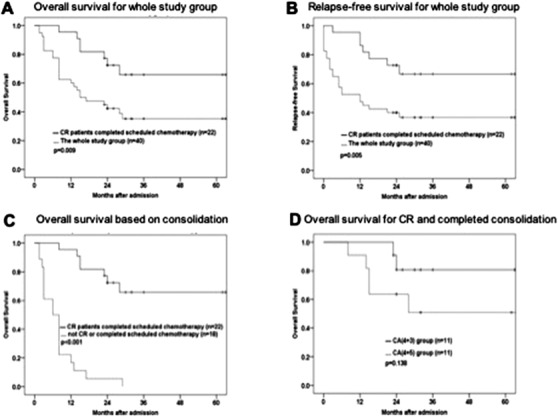
Outcomes in CA subgroups. (A) Overall survival for whole study group; (B) RFS for whole study group; (C) overall survival for patients completed scheduled chemotherapy or not; (D) overall survival for patients achieved CR and completed scheduled chemotherapy

We further analyzed the survival of the 22 CR patients, with 11 patients in each subgroup, who completed the scheduled three courses of consolidation regimens. The expected 5 years OS and relapse‐free survival (RFS) of these patients were 64%, higher than those did not achieve CR, or did not complete the scheduled consolidation chemotherapy (*P* < .001) (Figure 3A‐C). In the CA4+3 group, nine patients were alive by the end of follow‐up, including one patient who underwent HSCT. The median follow‐up time of the 11 CR patients in the CA4+3 group was 30 (23‐66) months. The expected 5 years OS and RFS were 78% and 78%, respectively (Figure 3D). In the CA4+5 group, four patients received Allo‐HSCT but two of them died of severe complications. Six patients were alive by the end of follow‐up (24 months, range 8‐64 months). The expected 5 years OS and RFS were 51% and 51%.

### 3.4 Adverse events

The CA regimen was generally safe and had reversible adverse effects. All patients attained grade IV myelosuppression, but no patients died in the first course of induction chemotherapy (Table 2). The WBC elimination and platelet recovery were comparable between the CA4+3 and CA4+5. Neutrophil and platelet recovery took a median time of 17 and 16 days in the CA4+3 group, faster than that in the CA4+5 group which were both 20 days (*P* < .001). Among the nonhematological adverse events, grade III‐IV infections were most common. Infections emerged in 16 patients (40%) during the myelosuppression. Ventricular premature beat and grade II hemorrhagic cystitis were recorded in one patient. The lesions to liver, kidney, and skin were slight (grade I). No cardiac toxicity was reported by the end of follow‐up. The incidence of nonhematologic adverse events was similar between the two groups.

**TABLE 2:** The adverse events of CA regimen

Adverse event (AE)	CA4+3	CA4+5	*P* value
Hematologic AE [d, M (range)]			
The nadir of WBC (× 10⁹/L)	0.1 (0.01‐1.05)	0.095 (0.02‐0.98)	.076
The day of ANC recovery (>0.5 × 10⁹/L)	17 (10‐20)	20 (16‐36)	<.01
The day of platelet (PLT) recovery (>20 × 10⁹/L)	16 (12‐34)	20 (14‐36)	<.01
Nonhematologic AE (%)			
Infection pharyngeal	1(5)	1(5)	
The upper respiratory tract	3(15)	0(0)	
Lungs	10(50)	10(50)	
Intestinal	11(55)	11(55)	
Crissum	0(0)	2(10)	
Subcutaneous tissue	0(0)	1(5)	
Fever of unknown origin	0(0)	1(5)	
Liver function lesion	0(0)	1(5)	
Rash	1(5)	4(20)	
Oral ulcer	1(5)	1(5)	
Arrhythmia	0(0)	1(5)	
Hemorrhagic cystitis	0(0)	1(5)	

## 4 DISCUSSION

The regimen of DA(3+7) has long been used as a standard induction chemotherapy for AML patients, and efforts have been made to improve the CR rate by escalating the dosage of daunorubicin. The CR/CRi rates of induction chemotherapy using daunorubicin 60–90 mg/m^2^ for 3 days were 66‐68% [10]. Another research reported that the patients who received daunorubicin (50 mg/m^2^ daily for 5 days) or idarubicin (12 mg/m^2^ for 3 days) achieved a similar CR rate (77.5% vs 78.2%) [16]. In this study, we evaluated the efficacy of an anthracycline‐free regimen in treating newly diagnosed adult AML. Our results demonstrate that the induction regimen of CTX and Ara‐C was efficacious for AML patients. With one course of induction, the CA regimen achieved a CR/CRi rate of 80%, similar to that of the DA regimen. Patients who did not respond to CA treatment also did not benefit from DA regimens.

Disease would relapse in nearly all CR patients without proper consolidation treatment [21]. The same results were observed in this study. Patients who gave up consolidation chemotherapy relapsed and died within a few months, thus led to a poor OS. In patients younger than 60 years who received intense chemotherapy but not followed by Allo‐HSCT, the reported 10 years survival and disease‐free survival (DFS) was only 16.6%. Patients elder than 60 years showed even worse DFS [22]. Unexpectedly, patients in our study who completed the consolidation chemotherapy showed an expected 5 years OS and RFS reached to 64%, higher than that of traditional treatment [9, 10, 23]. But this result needs to be interpreted cautiously due to the limited number of patients. We did not assess the efficacy and survival of patients at different risk groups due to the limited case number. Patients in this study were relatively young (median 44.5 years old) and might respond well to the CA regimen. Future studies with a larger cohort of AML patients may address the heterogeneity of chemosensitivity to CA induction therapy within different risk stratification and among different ages and determine the unfavorable factors affecting CR and OS.

High‐dose Ara‐C may overcome cytarabine resistance in leukemic blasts and has been used successfully as a salvage regimen for AML as well as in postremission therapy [24]. However, studies showed that using Ara‐C at > 1 g/m^2^ during induction did not increase efficacy in newly diagnosed AML [25, 26], but increased drug toxicity (NTR230) [27]. Therefore, we adopted Ara‐C 1 g/m^2^ for induction chemotherapy in our study. CTX is an inactive prodrug that requires enzyme and chemical activation. Considering severe adverse events to heart or liver that often occur in HSCT patients using CTX 60 mg/kg for preconditioning, we reduced the dosage to 20 mg/kg per day. The regimen was generally safe, and we did not observe severe nonhematological toxicity. All patients survived using the current dose of Ara‐C and CTX, indicating its safety as an induction therapy. Importantly, the CA regimen shortened the chemotherapy duration from traditional 7 days to 4–5 days, which significantly reduced the patients’ torture both physically and mentally.

One concern of the hematologic toxicity of the induction regimen is the duration of agranulocytosis that resulted in life‐threatening infections. It usually takes 29 ± 10 days for neutrophile to recover to > 0.5 × 10^9^/L in traditional chemotherapy [9, 10, 23]. The median Absolute Neutrophil Count (ANC) recovery time of the CA regimen was 18 days, with faster recovery in the CA4+3 group. This may attribute to the rapid obliteration of the leukemic cells due to the higher cumulative drug concentration and sustained long‐lasting plasma concentration of Ara‐C daily used in the CA4+3 group than that in the CA4+5 group.

In this study, we first time demonstrate that the CA regimen could achieve high CR rate and long‐term survival in newly diagnosed AML, with short chemotherapy duration and fast ANC recovery. Our results indicate that the CA induction regimen be a good alternative induction option for AML patients. CTX has clear potential in treating AML, and thus further investigation is warranted using the nonanthracycline drugs as a substitution for the induction chemotherapy regimen.

## CONFLICTS OF INTEREST

The authors declare that they have no conflict of interest.

## AUTHOR CONTRIBUTIONS

Liu Q designed the study, enrolled patients, analyzed data, and wrote the manuscript. Gao H performed statistical analysis and drafted the manuscript. Li J, Hu Y, and Wu L enrolled patients and edited the manuscript. Zhao X and Li S analyzed data and edited the manuscript.

## DATA SHARING STATEMENT

The data that support the findings of this study are available on request from the corresponding author. The data are not publicly available due to privacy or ethical restrictions.
